# Retrospective evaluation of resident pharmacists’ services in a liver intensive care unit: a single-center experience

**DOI:** 10.3389/fphar.2025.1583818

**Published:** 2025-09-18

**Authors:** Huijie Meng, Zhaoshuai Ji, Zhenyu Zhang, Yuanyuan Liu, Fang Li, Jing Tang, Guangmeng Nie

**Affiliations:** ^1^ Department of Pharmacy, Beijing Tsinghua Changgung Hospital, School of Clinical Medicine, Tsinghua Medicine, Tsinghua University, Beijing, China; ^2^ Department of Livre ICU, Beijing Tsinghua Changgung Hospital, School of Clinical Medicine, Tsinghua Medicine, Tsinghua University, Beijing, China; ^3^ Beijing Tsinghua Changgung Hospital, School of Clinical Medicine, Tsinghua Medicine, Tsinghua University, Beijing, China

**Keywords:** resident pharmacist, intensive care unit, clinical pharmacy, antimicrobial stewardship, cost-effectiveness

## Abstract

**Background:**

The engagement of clinical pharmacists in ward-based care remains limited in our hospital. This study explores a novel pharmacist working model (resident pharmacists) and evaluates its effectiveness to provide evidence for optimizing pharmaceutical services in intensive care units (ICUs).

**Methods:**

From February 2024 to January 2025, resident pharmacists were embedded within the Liver ICU as part of a pilot program, delivering multidisciplinary pharmaceutical services including clinical care, teaching, research, and management. Specialized interventions targeting pediatric and infectious disease populations were implemented based on departmental needs. A retrospective analysis was conducted over 12 months to assess the impact of resident pharmacists on pharmaceutical indicators, service quality, and staff satisfaction; key outcome measures included cost reduction, prescribing appropriateness, and antibiotic use metrics.

**Results:**

All pharmaceutical management metrics met institutional standards in 2024-2025, including Antibiotic Use Density (AUD; 112.30 vs. threshold 120.47), antimicrobial usage rate (78.68% vs. 88.54%), and proton pump inhibitor prescription rationality (100%). Pharmacists conducted 78 clinical consultations (65.38% multidisciplinary), addressed 132 medication inquiries, developed two clinical guidelines, and achieved full satisfaction scores from medical staff. The average drug cost per patient decreased by 20.05% (RMB 28,806 vs. 36,028 in 2023), with drug cost proportion declining from 30.85% to 23.59%.

**Conclusion:**

By deeply participating in various aspects of drug use in the wards, including medical work, management, scientific research cooperation, continuing medical education, resident pharmacists provided more high-quality pharmaceutical services and promote the improvement of pharmaceutical quality control indicators. Resident pharmacists have become important members of the medical team responsible for medication safety and optimizing treatment plans.

## 1 Introduction

Clinical pharmacists are playing an increasingly pivotal role within healthcare teams, with their responsibilities expanding beyond traditional medication dispensing to encompass core aspects of clinical care. Within cardiac care teams, clinical pharmacists have become vital members of the interdisciplinary team, actively participating in the development of medication regimens and implementing interventions to reduce medication-related problems and adverse drug reactions (Huynh et al., 2023; Suman and Pravalika, 2023). In diabetes management, clinical pharmacists significantly improve patients’ glycemic control through medication adjustments, the establishment of diabetes care centers, and the provision of health counseling (Theivasigamani and Palaniappan, 2024) Furthermore, clinical pharmacist interventions enhance patient safety by addressing medication-related issues and help curb avoidable healthcare costs (Sun et al., 2023). Pharmacists’roles in intensive care units (ICUs) are increasingly clinical. A 2024 cross-sectional study on ICU staffing in US hospitals showed that 92.6% of ICUs are equipped with clinical pharmacists (Gershengorn et al., 2024). Studies (Lee et al., 2019) confirm that ICU pharmacists improve care quality and reduce costs.

Currently, gaps in role perception remain a significant challenge. Studies indicate that insufficient understanding of clinical pharmacists’ responsibilities among physicians and nurses may hinder their effectiveness (Crafford et al., 2023). Within South Africa’s public healthcare system, clinical pharmacists encounter challenges related to low professional recognition and resource constraints as they transition from traditional dispensing to clinical roles (Crafford et al., 2025). Enhancing interdisciplinary communication can improve the acceptance rate of clinical pharmacists’ recommendations, thereby optimizing medication therapy safety (Mawardi et al., 2025). In China, despite widespread adoption of clinical pharmacists, their impact remains suboptimal. In 2023, the National Health Commission advocated for resident pharmacists to address this gap. The resident pharmacist is a new exploration of the pharmacist service model in China in recent years. The key difference between this working model and traditional clinical pharmacy practice is that it brings pharmacists closer to the clinical frontlines and clearly defines the duties of ward-based pharmacists within clinical departments.

Our hospital is a tertiary teaching hospital affiliated with Tsinghua University, where our pharmacists have been providing inpatient monitoring and outpatient medication consultation services. In early 2024, our hospital was selected as a pilot hospital for resident pharmacist work. As a national pilot site, our hospital implemented resident pharmacists in three departments, including the Liver ICU (LICU). This article introduces the working model of ICU resident pharmacists currently established in our hospital, and conducts a retrospective analysis of the service effectiveness of ICU resident pharmacists in 2024-2025, aiming to provide a reference for the work of other ICU pharmacists.

## 2 Methods

### 2.1 Study design and setting

This single-center retrospective study analyzed data from the LICU at Beijing Tsinghua Changgung Hospital (February 2024 - January 2025). The LICU manages post-transplant and complex surgical patients, with resident pharmacists focusing on:

1. Anti-infective therapy for immunosuppressed patients;
2. Pediatric dosing (<1 year);
3. Dose adjustments in hepatic/renal dysfunction.

#### 2.1.1 Data source

All data were retrospectively extracted from our hospital’s Clinical Information System (CIS), encompassing: 1 Antimicrobial stewardship metrics; 2 Pharmacoeconomic indicators; 3 Medication appropriateness rates.

#### 2.1.2 Inclusion criteria

Departmental data from the LICU spanning February 2024 to January 2025 were included, with data attribution based on medication order initiation units rather than discharging departments.

#### 2.1.3 Exclusion criteria

As this study utilized aggregated departmental-level data without individual patient identifiers, no exclusions were applied.

#### 2.1.4 Statistical analyses

Statistical software (SPSS 23.0) was used for data analysis. For each variable, continuous data were summarized as mean ± standard deviation or median with interquartile range, while categorical data were presented as counts and percentages. Student’s t-test or Mann-Whitney U test was performed to compare quantitative variables, while chi-square test or Fisher exact test was performed to compare categorical data. Statistical significance was defined as p < 0.05.

### 2.2 Interventions

**FIGURE 1:**
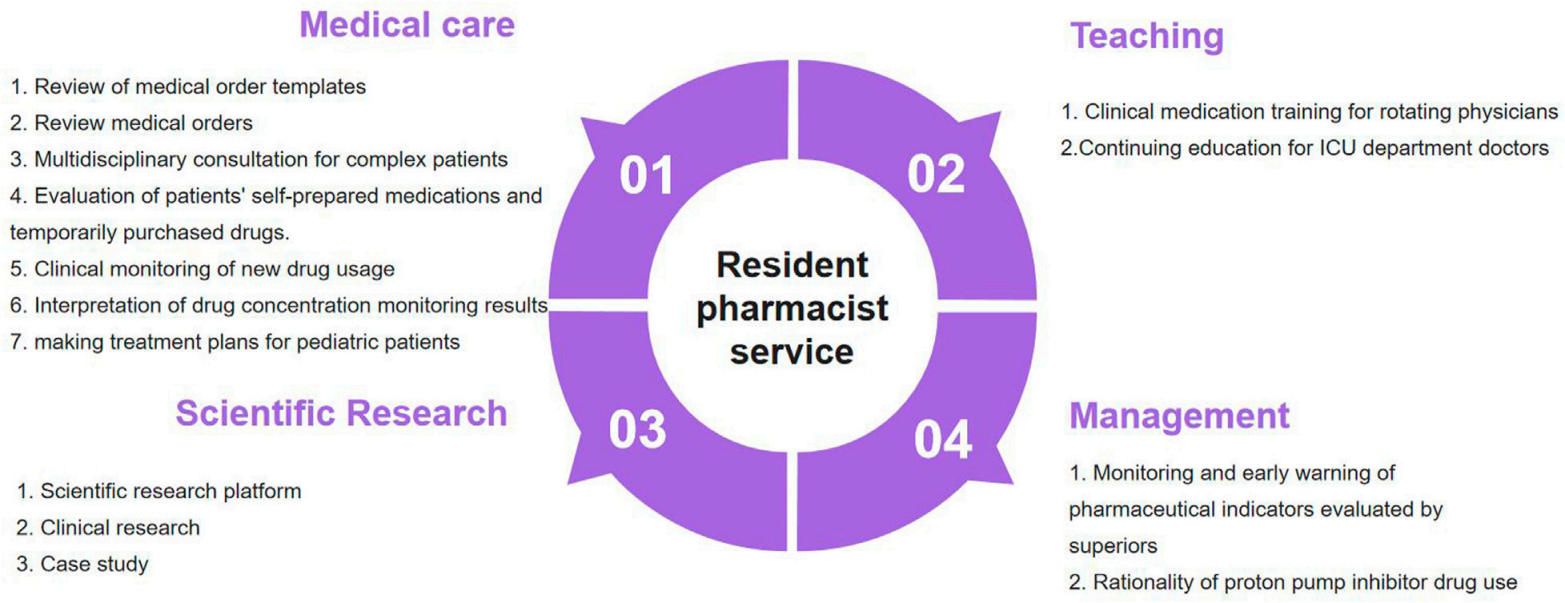
The content of pharmaceutical services provided by ICU resident pharmacists.

The LICU at our institution operates a 16-bed tertiary care facility specializing in perioperative management of transplant recipients (hepatic, renal, and intestinal allografts) and complex surgical cases involving hepatic resection and pediatric hepatobiliary procedures. Unlike traditional clinical pharmacists, resident pharmacists were actively involved in comprehensive pharmaceutical activities, including but not limited to medication monitoring, clinical rounds, and collaborative decision-making,becoming communicators and managers of rational drug use in clinical departments. After preliminary communication with the clinical department, LICU pharmacists carried out resident pharmacist had worked from all dimensions including medical care (40%), teaching (25%), scientific research (20%), and management (15%). The job responsibilities of resident pharmacists are illustrated in Figure 1.

The study was implemented through the following steps:

• Full communication with physicians and nurses in clinical departments to understand their medication needs;
• Formulating a service plan for resident pharmacists;
• Providing specialized pharmaceutical services based on patient characteristics;
• Developing a questionnaire on medical staff satisfaction;
• Providing pharmaceutical services focusing on medical treatment, teaching, scientific research, and management;
• Evaluating the effectiveness of pharmacists’ work.

#### 2.2.1 Pharmaceutical care for patients

In medical work, resident pharmacists had similar roles to clinical pharmacists, but they provided more comprehensive monitoring and require more time. Resident pharmacists actively fulfilled their duties and worked closely around patient monitoring included: 1. Reviewing medical order templates and revising inappropriate usage; 2. Daily reviewing of inpatient medical orders 3. Clinical consultations, offering professional medication advice to medical staff; 4. Engaging in departmental case discussions, focusing on anti-infective regimens and dosages for special populations; 5. Answering to medical staffs’ questions about medication administration. Resident pharmacists had joined the medical team through various forms of participation, establishing an efficient and seamless interactive mechanism and working mode.

Based on the characteristics of the department, the pharmacists provided some specialized pharmaceutical services, including pediatric transplant pharmacotherapy consult service and antimicrobial stewardship for multidrug-resistant organisms. As integral members of the Pediatric Liver Transplant Multidisciplinary Team, clinical pharmacists provide perioperative pharmacotherapy optimization through: age-specific dose adjustment algorithms; evidence-based off-label use documentation; therapeutic drug monitoring protocols. Patients in the ICU after transplantation often suffer from abnormalities in liver and kidney function, as well as immunosuppression. The ICU is a department with a high detection rate of multidrug-resistant bacteria. In special physiological states, the treatment plans for multidrug-resistant bacteria often faces more contraindications and fewer treatment drug options. Pharmacists focused on the medication audit of patients in this group, and participated in the treatment for multidrug-resistant bacteria in special populations through ward rounds, case discussions, clinical consultations, etc.

#### 2.2.2 Pharmaceutical indicator management

Beyond direct clinical care, performance monitoring of pharmacotherapy metrics mandated by regulatory authorities represents a crucial pharmaceutical service provided by clinical pharmacists. Our department implemented a structured reporting system where comprehensive pharmaceutical indicator analyses are delivered to the ICU director twice monthly (mid-month and month-end). This systematic approach enables clinical teams to monitor guideline adherence patterns and implement corrective measures promptly. The monitored parameters encompass:

1. Antimicrobial usage rates
2. Antibiotic Use Density (AUD) - calculated as Σ(defined daily doses × drug weight)/100 patient-days.
3. Prescription rationality rate of proton pump inhibitors;
4. Average drug cost per inpatient visit;
5. Drug-to-total cost ratio

#### 2.2.3 Educational initiatives

Clinical pharmacists play a dual educational role, conducting evidence-based pharmacotherapy training for rotating residents while providing continuing medical education (CME) programs for attending physicians. Through interactive case-based seminars and therapeutic guideline workshops, we standardize prescribing behaviors and enhance medication safety awareness. Core curriculum components include:Antimicrobial stewardship in critical care; High-alert medication management; Real-time literature updates on newly approved agents.

#### 2.2.4 Research collaboration

A multidisciplinary research consortium was established between the Liver ICU and Department of Pharmacy, leveraging clinical informatics to advance pharmaceutical sciences. Current research foci include:

1. Population pharmacokinetic modeling of tacrolimus in transplant recipients
2. Artificial intelligence-driven early warning systems for nephrotoxic medications
3. Quality improvement initiatives in aerosolized drug delivery

### 2.3 Evaluation metrics

#### 2.3.1 Antimicrobial stewardship

1. AUD < 120.47 (dept threshold)
2. Antibiotic use rate < 88.54% (dept threshold)

#### 2.3.2 Medication appropriateness

1. Antimicrobial Appropriateness Rate ≥ 96%
2. Proton Pump Inhibitor (PPI) Appropriateness Rate ≥ 96%

#### 2.3.3 Provider satisfaction

Rising satisfaction among physicians/nurses with pharmacists.

#### 2.3.4 Pharmacoeconomics

1. Reduction in per-admission medication costs
2. Decrease in the medication-to-total-medical-cost ratio

## 3 Results

From February 2024 to January 2025, pharmacists monitored 1046 patients, and through medication advice, pharmaceutical consultation, departmental training, the medication awareness of medical staff was improved. There were improvements in ICU drug indicators, drug costs, and satisfaction.

### 3.1 Antimicrobial stewardship

Antibiotic Use Density (AUD) emerged as the pivotal antimicrobial stewardship metric in critical care settings. Following clinical pharmacist integration into the Liver ICU team, a 32% reduction in empirical antibiotic prescribing was achieved (2023–2024). Key performance indicators demonstrated:

• AUD: 112.30 (vs. institutional target ≤ 120.47)
• Antimicrobial prescribing rate: 78.68% (vs. threshold 88.54%)
• 12 months sustained compliance with stewardship benchmarks

### 3.2 Clinical impact

#### 3.2.1 Prescribing appropriateness

Through proactive order auditing and biweekly therapeutic workshops, significant improvements were observed:

• Proton pump inhibitor appropriateness: 100%
• Antimicrobial regimen rationality rate: 97.41%

The evaluation criteria for proton pump inhibitors strictly follow high-risk factors, drug selection, dosage form, frequency of administration, and other aspects.

#### 3.2.2 Pharmaceutical consultation

**FIGURE 2:**
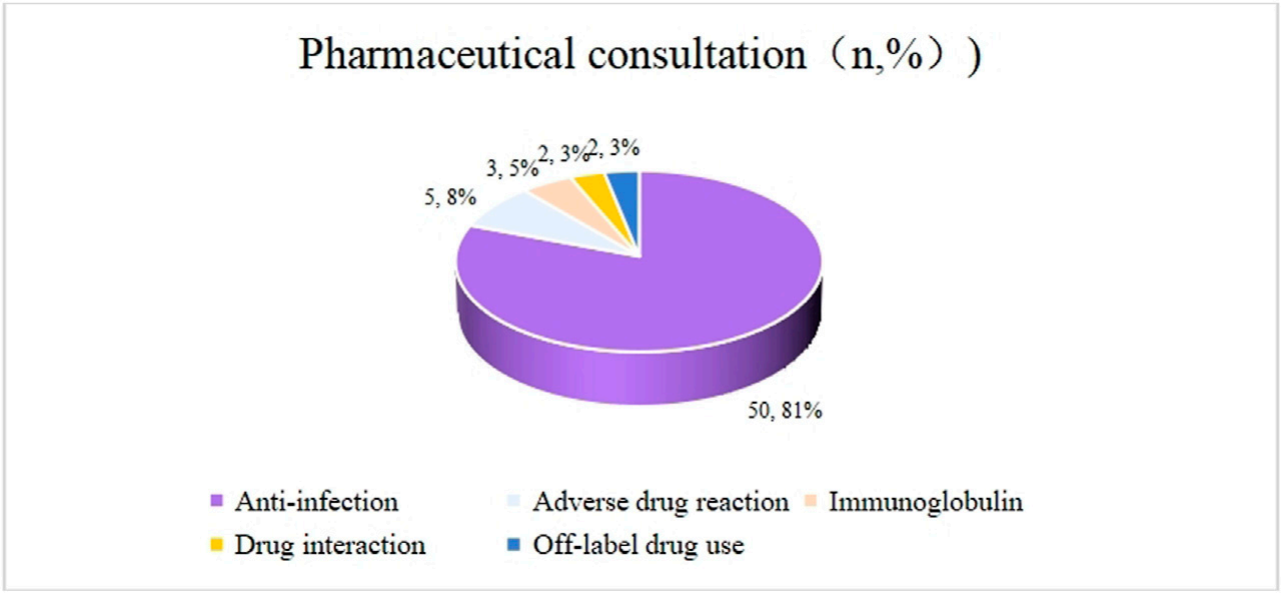
Question types of pharmaceutical consultations in 2024.

Pharmaceutical consultation is an important form for pharmacists to participate in clinical treatment. From February 2024 to January 2025, pharmacists conducted 78 consultations, among which 27 cases (34.61%) were routine consultations and 51 cases (65.38%) were multidisciplinary consultations. The top three issues related to medication in the consultations were anti-infective drug treatment (53 cases, 68%), evaluation adverse drug reactions (11 cases, 14%), and drug interaction (5 cases, 6%). The specific situation is shown in Figure 2, and the adoption rate of the consultation was 100%.

#### 3.2.3 Medication standardization

As part of ongoing quality improvement initiatives, clinical pharmacists developed two evidence-based therapeutic guidelines addressing critical medication challenges in specialized patient populations. The first guideline established standardized protocols for antimicrobial stewardship in liver transplantation recipients during the perioperative period, while the second focused on optimized pharmacotherapy strategies for pediatric patients. These documents were created following multidisciplinary consultations and rigorous review of current clinical evidence.

### 3.3 Cost reduction

Comparative pharmacoeconomic analysis revealed substantial optimization in medication-related expenditures for critically ill patients. In 2024, the mean pharmaceutical cost per admission in the Liver Intensive Care Unit (LICU) decreased significantly to ¥28,806 (USD 2,996), representing a 20.05% reduction from the 2023 baseline of ¥36,028 (USD 4,997). This reduction exceeded institutional cost-containment benchmarks while maintaining therapeutic efficacy. Concurrently, the medication expense proportion within total hospitalization costs demonstrated a notable decline from 30.85% (2023) to 23.59% (2024), reflecting improved resource allocation and enhanced adherence to antimicrobial stewardship protocols. The department exhibited statistically significant decreases in 2024 versus 2023 for: (a) per-admission medication costs, (b) medication-to-total-cost ratio, and (c) length of stay (p < 0.01 for all; Table 1).

**TABLE 1:** Healthcare cost burden and hospital length of stays among patients.

Group	All patients (n = 2,244)	Medication costs (¥10,000)* [M (P25,P75)]	Medication-to-total-medical-cost ratio (%) [M (P25,P75)]	Length of stay (days) [M (P25,P75)]
2023	1,113	1.73 (0.72, 5.11)	19.93 (12.81,31.03)	16 (11, 23)
2024	1,131	1.37 (0.55, 3.64)	16.10 (10.26,26.08)	15 (10, 21)
Z value		−4.667	−7.225	−3.120
P value		<0.001	<0.001	0.002

*All costs are Chinese Yuan, per 10 thousand Yuan.

## 4 Discussion

### 4.1 Antimicrobial stewardship in critical care settings

Antimicrobial drug management is one of the critical issues in the ICU, and it has always been a significant aspect of pharmacists’ work. Studies have shown that, pharmacists can be involved in Antibiotic stewardship programmes (ASPs) to facilitate the appropriate antibiotic use. Meta-analysis revealed that ASP implementation in critically ill neonates was significantly associated with a 23% reduction in the overall antibiotic use rate (Lee and An, 2022). A retrospective study showed that,Pharmacist-driven interventions significantly reduced the days of therapy (DOT) for carbapenems and days (Nakano et al., 2025). The management of antibacterial drugs in our hospital has consistently been excellent. The hospital has established an antibacterial drug management working group, comprising doctors, pharmacists, laboratory departments, information offices, teaching departments, and more. This group continuously undertakes ASP projects, which involve setting departmental thresholds for antibacterial drugs in clinical departments.

After our hospital’s resident pharmacists commenced their work in the LICU, they contributed to meeting the antibacterial drug standards in the LICU and reducing the consumption of antibacterial drugs by providing training to doctors and nurses, reviewing medical orders, maintaining timely communication, participating in consultations, and engaging in case discussions. One detail is that in November, the AUD rose and exceeded the departmental threshold. Upon analysis, we identified the primary reason as seasonal fluctuations in patient admissions, with winter being a high-incidence period for infectious diseases. Additionally, we examined the rationality of medication use and found no evident irrationalities. In December, to ensure that the annual targets were met, we monitored indicators every 10 days, rigorously reviewing combined medication and postoperative prophylaxis courses. Consequently, the AUD decreased in December compared to November, and all annual targets were achieved. This retrospective analysis highlights the critical role of pharmacist interventions in optimizing antimicrobial stewardship. Additionally, it serves as a valuable reference for other ICU pharmacists as they undertake antimicrobial work.

### 4.2 Economic benefits of pharmacist work

Pharmacist-led interventions demonstrated significant cost-saving potential in drug expenditure. In a Dutch study evaluating the impact of clinical pharmacy interventions on the number of ADEs, the cost–benefit was estimated at €119–136 per accepted intervention (Bosma et al., 2018). A recent scoping review reported a median cost–benefit of £6.42 per £1invested in pharmacy services (Crosby et al., 2023). Economic analyses have consistently indicated a high return on investment, with the predicted cost-avoidance to pharmacists’ salary ranging from $3.3:1 to $9.6:1 (Wei et al., 2024). In our study, following 12 months of pharmacist work, there were significant decreases in both average medication cost per patient and medication-to-total-cost ratio in 2024 compared to 2023. These figures demonstrate a significant decrease in both the absolute and relative values of patients’ drug costs. While the reduction in drug costs is attributed to the collaborative efforts of doctors and pharmacists, the magnitude of the decrease is substantial, sufficiently indicating that pharmacists’ work can contribute to lowering drug costs.

### 4.3 The work content carried out by ICU pharmacists

The ultimate goal of pharmacists’ work is to benefit patients. ICU pharmacists at a tertiary hospital in the United Arab Emirates recommended 1,004 intervention suggestions to 200 patients. Patients who received four or more intervention suggestions had a reduced cumulative risk of death within 90 days of ICU admission (Ali Hussain Alsayed et al., 2024). The European Society of Critical Care Medicine has spoken highly of the pharmaceutical professionals working in the intensive care unit. It is believed that the work of pharmacists can improve the clinical outcomes of patients in the intensive care unit. This improvement is primarily achieved through enhancing patient safety through pharmacovigilance expertise, optimizing the treatment drug regimen using pharmacology knowledge, and drug dose adjustments and therapeutic drug monitoring for patients with multiple organ failure. Our resident pharmacists are also engaged in the aforementioned monitoring tasks, ensuring that their work remains at the forefront of both international and domestic pharmaceutical development. For a comparison of the work contents between our hospital’s resident pharmacist service and European pharmacist service, please refer to Table 2.

**TABLE 2:** Comparison of the work between pharmacists in our hospital and European pharmacists.

Item	Ten reasons for the presence of pharmacy professionals in the intensive care unit	Does our hospital provide
1	Improve ICU patient and clinical outcomes	Not conducted
2	Expertise in pharmacovigilance improves patient safety	YES
3	Pharmacological expertise is vital for ICU medication optimisation	YES
4	ICU pharmacists understand drug dosing in multiple organ failure and therapeutic drug monitoring	YES
5	ICU pharmacy professionals are well placed to conduct medication reconciliation at transitions of care	NO
6	Pharmacy technicians support nursing colleagues and contribute to sustainable healthcare	NO
7	Pharmacy technicians are responsible for adequate drug delivery and stock supply	Part of the work
8	Clinical pharmacists have a lead in drug use research	YES
9	Clinical pharmacists contribute to education and delivery of evidencebased pharmacotherapy	YES
10	Clinical pharmacy services are associated with cost–benefit	Not conducted

### 4.4 Limitations

This study has several limitations that warrant acknowledgment. First, its single-center scope at our tertiary hospital restricts the generalizability of findings to institutions with different patient demographics, resource availability, or clinical practices. Second, the retrospective design introduces potential biases in data collection and limits our ability to establish causal relationships between pharmacist interventions and observed outcomes. Third, the lack of a control group prevents definitive attribution of improvements (e.g., reduced antimicrobial use, cost savings) solely to pharmacist activities, as concurrent quality initiatives or secular trends could contribute.

Future research directions should include:

• Multi-center prospective studies with matched control groups to validate causality and enhance generalizability;
• Long-term assessments of clinical outcomes (e.g., mortality, readmission rates) linked to pharmacist interventions;
• Economic evaluations comparing different pharmacist integration models.

## 5 Conclusion

The pilot project of resident pharmacists represents an exploration of a new working model for pharmacists serving in the clinical setting. The working model of resident pharmacists in our hospital’s intensive care unit has been preliminarily established. The resident pharmacists devoted more time and energy to clinical medication, delving deeply into the promotion of rational clinical drug use across multiple dimensions, including clinical medication, teaching, scientific research, and management. This dedication aims to enhance medical quality and ensure patient medication safety. Our exploration work also demonstrates that pharmacists, by joining the clinical treatment team and addressing clinical issues in depth, can ultimately enhance medical quality and improve the level of clinical rational drug use. This includes increasing the rational rate of drug use, improving antimicrobial indicators, and achieving significant savings in drug costs.
